# *DISC1* Conditioned GWAS for Psychosis Proneness in a Large Finnish Birth Cohort

**DOI:** 10.1371/journal.pone.0030643

**Published:** 2012-02-17

**Authors:** Liisa Tomppo, Jesper Ekelund, Dirk Lichtermann, Juha Veijola, Marjo-Riitta Järvelin, William Hennah

**Affiliations:** 1 Institute for Molecular Medicine Finland (FIMM), University of Helsinki, Helsinki, Finland; 2 National Institute for Health and Welfare, Helsinki, Finland; 3 Department of Psychiatry, University of Helsinki, Helsinki, Finland; 4 Vaasa Hospital District, Vaasa, Finland; 5 Café Ersatz, Bonn, Germany; 6 Department of Psychiatry, University of Oulu, Oulu, Finland; 7 Department of Epidemiology and Public Health, Imperial College, London, United Kingdom; 8 Medical Genetics Section, University of Edinburgh, Edinburgh, United Kingdom; University of Hong Kong, Hong Kong

## Abstract

**Background:**

Genetic evidence implicates the *DISC1* gene in the etiology of a number of mental illnesses. Previously, we have reported association between DISC1 and measures of psychosis proneness, the Revised Social Anhedonia Scale (RSAS) and Revised Physical Anhedonia Scale (RPAS), in the Northern Finland Birth Cohort 1966 (NFBC66). As part of the studies of this Finnish birth cohort genome-wide association analysis has recently been performed.

**Methodology:**

In the present study, we re-analyzed the genome-wide association data with regard to these two measures of psychosis proneness, conditioning on our previous *DISC1* observation. From the original NFBC66 sample (N = 12 058), 4 561 individuals provided phenotype and genotype data. No markers were significant at the genome-wide level. However, several genes with biological relevance to mental illnesses were highlighted through loci displaying suggestive evidence for association (≥3 SNP with P<10E-4). These included the protein coding genes, *CXCL3*, *KIAA1128*, *LCT*, *MED13L*, *TMCO7*, *TTN*, and the micro RNA *MIR620*.

**Conclusions:**

By conditioning a previous genome-wide association study on *DISC1*, we have been able to identify eight genes as associating to psychosis proneness. Further, these molecules predominantly link to the DISC1 pathway, strengthening the evidence for the role of this gene network in the etiology of mental illness. The use of quantitative measures of psychosis proneness in a large population cohort will make these findings, once verified; more generalized to a broad selection of disorders related to psychoses and psychosis proneness.

## Introduction

Disrupted in Schizophrenia 1 (*DISC1*) is one of the most promising candidate genes for schizophrenia with abundant supporting evidence from linkage, association, gene expression studies and animal models (for review see [1]). In order to follow-up the prior identification that three *DISC1* SNPs (rs821577, rs821633 and rs1538979) modify risk to schizophrenia and bipolar disorder dependent on interplay [2], we studied these SNPs in relation to four psychometric scales evaluated in the Northern Finland Birth Cohort 1966 (NFBC66). We noted that the three SNPs significantly affect population wide measures of psychosis proneness [3], significantly modifying scores of Revised Social Anhedonia Scale (RSAS) and Revised Physical Anhedonia Scale (RPAS) through interplay. RSAS and RPAS are characteristic symptoms of schizophrenia, in their severe forms they describe an individual's incapability to appreciate physical pleasure (physical anhedonia) or social contact (social anhedonia). Individuals scoring high in these dimensions are assumed to bear a behavioral endophenotype [4] that increases their risk to develop overt psychotic syndromes, and indeed follow-up investigations of the original cohorts have provided evidence of varying degree for the presence of these behavioral traits [5].

In addition to *DISC1*, a number of genes encoding proteins that interact with DISC1 have emerged as promising candidate genes for schizophrenia and other major mental illnesses [1]). As a result, the concept of the DISC1 pathway has been proposed. This hypothesis proposes that in addition to disruption of *DISC1*, the disruption of other genes in the pathways *DISC1* is involved in could also increase risk to mental illness [6]. One key study in this DISC1 pathway hypothesis was the identification of *NDE1*. The *NDE1* gene only came to light when we re-analyzed our genome-wide linkage data conditioned on a *DISC1* associating risk haplotype [7]. In that study, families with the risk allele of DISC1 and those without were treated as two separate sample sets hypothesizing that since locus heterogeneity certainly exists between families, this ascertainment strategy could provide some added power, through reducing this genetic heterogeneity, to identify genes involved in the molecular pathogenesis of schizophrenia. Although none of the identified conditioned linkage peaks reached significance, a number of defined peaks were observed at genomic regions previously strongly implicated in schizophrenia and/or bipolar disorder [7]. One such peak was at chromosome 16p13 close to NDE1 which in a follow-up analysis revealed significant association to schizophrenia in Finnish families ascertained for schizophrenia [7]. Since this observation, *NDE1* has been implicated in the etiology of schizophrenia in several studies world wide (for review [8]).

In the present study, we exploited the conditioned approach and performed a genome-wide association study (GWAS) on RSAS and RPAS dependent on the *DISC1* variants previously detected to associate with these traits. In the unconditioned genome-wide association study of the four psychometric scales, no significant association was detected to RSAS or RPAS (Ekelund et al 2011, in preparation). We hypothesized that through stratification based on the significant DISC1 variants, we are more likely to identify; due to increased homogeneity in the stratified samples; variants of small effect size that have direct biological relevance to psychosis proneness and potentially interact with *DISC1*.

## Results

A total of eight genome-wide association scans have been performed using different models and phenotypes. No markers reached the genome-wide significance level of P<5E-8, under any of the models tested. However, using the criteria of at least three SNPs (within a 300 kb genomic region) displaying P<10E-4, 18 clusters of suggestive evidence were observed (Figure S1).

**Figure 1:**
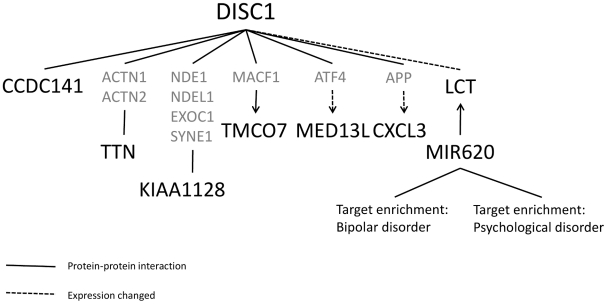
Illustration of how the identified molecules are relevant within the DISC1 pathway.

Both coding (Table S1) and non-coding genes were identified at these clusters. These were studied for relevance to the DISC1 pathway. A total of 24 genes and 2 micro RNAs were identified at the 18 loci. Of these, seven genes had prior evidence for involvement with the DISC1 pathway. Two genes were observed that directly interact with *DISC1*, *CCDC141* (2q31.2) which binds to the DISC1 protein [9] and LCT (2q21.3) which has been noted to have significantly increased expression in carriers of a certain *DISC1* genotype [10]. Three genes were noted to bind to previously known *DISC1* interactors, *TTN* (2q31.2) interacts with *ACTN1* and *ACTN2* [6], *KIAA1128* (FAM190B) (10q23.1) interacts with NDE1, NDEL1, EXOC1 and SYNE1 [11], while *TMCO7* (16q22.1) binds *MACF1* [12]. Additionally, two genes are regulated at the gene expression level by DISC1 binding partners, *CXCL3* (4q13.3,) by APP and *MED13L* (12q24.21) by *ATF4* [13], [14] (Table 1, Figure 1, Table S2).

**Table 1:** Summary of the associated genes with biological relevance for schizophrenia or the DISC1 pathway.

Chr	Position (kb)	Model	SNPs P<1E-4	Best SNP	Best SNP P-value	Number of genes	Number of miRNA's	Biologically relevant targets
2q21.3	136126–136361	RPAS risk	5	rs4954633	1.84E-06	3	0	*LCT*
2q31.2	179169–179462	RPAS covariated	3	rs2046778	1.67E-05	2	0	*CCDC141*, *TTN*
4q13.3	75104–75219	RSAS covariated	3	rs9131	3.43E-05	2	0	*CXCL3*
10q23.1	86142–86298	RPAS risk	4	rs10509494	5.36E-05	1	0	*KIAA1128*
12q24.21	114894–115222	RPAS neutral	3	rs11615689	2.24E-06	1	1	*MED13L*, *MIR620*
16q22.1	67640–67703	RSAS protective	4	rs3785079	1.82E-05	2	0	*TMCO7*

Abbrevioations Chr: chromosome; kb, kilobase (according to NCBI build 36.1).

In addition to the coding genes, the two miRNAs identified within the cluster regions were studied further through *in silico* techniques. This focused on the identification of genes that are reported to be targeted by the micro RNAs, and then testing these target genes for biological, functional or pathway enrichment. *MIR620* has predicted target sites in 896 genes with the most statistically enriched category of these genes in IPA being for genes previously implicated in bipolar disorder (66 molecules; P = 1.93E-05). The second most enriched category was psychological disorders (93 molecules; P = 2.19E-05) (Table S3). A number of the genes in these categories, including LCT, have been previously directly implicated in the etiology of schizophrenia and psychosis (Figure S2). The 1 093 predicted target sites of MIR128-1 did not display principal enrichment in pathways related to central nervous system or mental illnesses (Table S4). The most significant categories were related to cancer (histiocytic sarcoma, P = 6.03E-05) while the most significant category related to nervous system development ranked as 158^th^ and was “size of forebrain” (2 molecules, P = 4.50E-03).

## Discussion

In this study we have re-analyzed our GWAS data for psychosis proneness measures in a large Finnish birth cohort. Performing *DISC1* conditioned GWAS for the quantitative traits Revised Social Anhedonia Scale (RSAS) and Revised Physical Anhedonia Scale (RPAS). No genome wide significance was observed. However, six loci displayed suggestive evidence for association, in the form of association clusters, and biological relevance. These loci were on chromosomes 2q21.3 (*LCT*), 2q31.2 (*CCDC141*, *TTN*), 4q13.3 (*CXCL3*), 10q23.1 (*KIAA1128*), 12q24.21 (*MED13L*, *MIR620*), and 16q22.1 (*TMCO7*).

The majority of the identified protein coding genes are yet to be fully characterized, *CCDC141*, *KIAA1128*, *CXCL3* and *TMCO7*. However, the proteins of these genes have been previously linked to the DISC1 pathway through binding *DISC1* itself or one of its interacting partners [6], [10]–[14]. Of those genes that have been further characterized *MED13L*, whose expression levels are regulated by the *DISC1* binding partner *ATF4*, has been implicated in brain and heart development [15]. The *TTN* gene codes for a protein expressed in striated and cardiac muscles which regulates the length of sarcomeres [16], [17], while the *LCT* gene is well known to encode the enzyme that metabolizes lactose. This latter observation initially provokes an obvious question, since *LCT* has been previously suggested to be an indicator for population stratification, due to a large variation of allele frequencies across different populations [18]. However, in a previous study, it was shown that within this sample there was no evidence for population stratification [19]. Further, if population stratification was present we would expect to notice non-normally distributed QQ plots. We observed no notable deviation from the significance level expected by chance in this study (Figure S3).

Despite the obvious question that the identification of *LCT* brings, it should not be a completely unexpected observation. *LCT* could legitimately have a role in the etiology of schizophrenia. Galactose produced through LCT has been implicated in neuronal development, learning and memory [20]. 2-deoxy-D-galactose has been implicated in long term potentiation [21], thus it is closely related to learning and memory functions [22]. Further, *LCT* has displayed evidence for association with schizophrenia in a genome-wide association study [23].

In addition to the protein coding genes identified, we noted two micro RNAs within our regions of interest. Further analysis through *in silico* techniques showed that molecules predicted to be targets of MIR620 are significantly enriched for genes previously implicated in bipolar disorder and nervous system development. This suggests that *MIR620* could play an important role in the brain and potentially in the etiology of mental illnesses through dysregulation of numerous genes involved in the normal functioning of the brain. It should also be noted that, *LCT* is one of the predicted target molecules of MIR620. It had previously been shown that expression levels of *LCT* are correlated with *DISC1* genotype [10]. The expression levels of *LCT* significantly increased in individuals carrying a DISC1 variant that also correlated with reduced DISC1 gene expression [10]. These provide further evidence that *LCT* could play a role in major mental illness. A role for MIR128-1 in neurological and psychological disorders has been suggested, potentially through Reelin signaling in neurons [24]. Thus, it is possible that *MIR128*-1 has a role in the etiology of mental illness. However, in our analysis of target enrichments the first biological function relating to the brain is the 158^th^ enriched category. Therefore, we can not lend support to this hypothesis.

In this study we have taken an exploratory approach to identifying new candidate genes in genetic studies of psychosis. Pathway analysis has guided the identification of genes of interest in regions displaying suggestive association evidence. However, the real strength of the approach lies in the fact that it identifies biologically plausible candidate genes that would not have been detected without conditioning the data on a previous finding. Naturally, the approach is predominantly hypothesis generating, therefore the genes identified here should be verified in other samples for a role in mental illness. Yet, the presence of several associating SNPs within the regions of interest and the similar effects seen on both RSAS and RPAS add internal support for the involvement of these loci in the psychosis proneness. The use of a quantitative measure of psychosis proneness assessed in a large population cohort already makes the findings more generalized to a broad selection of disorders related to psychoses and psychosis proneness. That the effects of these molecules were not observed in the unconditioned analysis, suggest that these molecules may have an effect dependent on *DISC1*, further increasing the importance of the *DISC1* related pathway in the etiology of mental illness.

## Materials and Methods

### Study sample

The Northern Finland Birth Cohort 1966 (NFBC66) sample has been described in detail elsewhere [3]. The study was started in 1965 in the two northernmost provinces of Finland (Oulu and Lapland). Data on the individuals born into this cohort and on their mothers and fathers were collected from the 24th gestational week. Altogether, 12 231 children were born into the cohort, and 12 058 of them were live born [25]. The cohort has been supplemented by data collected by postal questionnaires at the ages of 1, 14, and 31 years, by hospital records and national register data, and by a physical examination at the age of 31 years. The phenotypic data analyzed in this study was included in a questionnaire offered to individuals at the 31-year follow-up. A total of 4651 individuals completed the questionnaire and provided a DNA sample along with written informed consent [3].

The scales that were included in the present study were the scales with which we previously detected association to DISC1. These scales were the Revised Social Anhedonia Scale (RSAS) [26], [27], and the Revised Physical Anhedonia Scale (RPAS) [28]. The psychometric properties of these scales in this specific setting have been previously reported [29], [30]. Previously it has been shown that social anhedonia, after controlling for the effects of the other psychosis proneness measures, independently and most clearly predicted later schizophrenia-spectrum personality disorders, social dysfunction and poor quality of relationships [31]. The Physical Anhedonia Scale was suggested to be a less effective tool for estimation of psychosis proneness [5]. However, in recent reports both physical anhedonia and social anhedonia have shown to be adequate indicators for later schizophrenia spectrum disorders [32] and psychosis [33].

The sample was stratified based on genotypes for SNPs under interplay that had previously been identified as associating to RSAS and RPAS. The minor allele of SNP rs821577, independent of the other markers, was significantly associated with higher scores on RSAS and RPAS [3]; that is individuals carrying this minor allele expressed significantly more anhedonic features. This SNP previously displayed evidence for association through increasing risk to bipolar disorder [2]. Thus, this variant is referred to as the risk variant. Marker rs821633 displayed a significant score reducing effect on these scales when conditioned on the absence of the minor alleles at rs821577 and rs1538979 [3]. This combination of alleles was also previously shown to reduce risk to both schizophrenia and bipolar disorder [2]. Thus, this combination of alleles is referred to as protective. Individuals carrying neither of these alleles were considered as carrying a neutral genotype. The number of individuals that carried risk, protective and neutral variants were 3 054, 962 and 545, respectively. Additionally, a non-stratified analysis was performed where *DISC1* variant status was used as a covariate. Due to inter-dependence between sex and the outcome measures, all the analyses were covariated for sex [34]. The phenotypic characteristics of the sample and the subsamples are presented in Table 2. Due to the test variables being slightly right skewed, logarithmic transformation was performed for each variable. After transformation, both RSAS and RPAS reached normality.

**Table 2:** Descriptive statistics on the outcome measures in the study sample.

Variable	RSAS				RPAS			
Sample	Whole	Risk	Neutral	Protective	Whole	Risk	Neutral	Protective
N	4556	3049	545	962	4556	3049	545	962
Mean^a^	9.5	9.64	9.52	8.97	15.12	15.28	15.04	14.53
95% CI of mean	9.34–9.66	9.44–9.84	9.02–10.03	8.62–9.33	14.91–15.32	15.02–15.53	14.40–15.68	14.08–14.97
SD	5.53	5.51	5.62	5.51	7.08	7.12	7.12	6.95

a Range for RSAS 0–40, range for RPAS 0–61. Abbreviations: RSAS: Revised Social Anhedonia Scale, RPAS: Revised Physical Anhedonia Scale, N: the number of individuals, CI: confidence interval, SD: standard deviation.

### Genotyping methods

Genotyping was performed using the Illumina Infinum Human CNV370-Duo marker set. SNPs with a success rate <95% or minor allele frequency (MAF) <0.05 and individuals with genotyping success rate <95% were excluded from the analyses. After quality controls 331 307 SNPs out of 339 054 SNPs and 5 251 individuals were included in the analyses. Out of these individuals, phenotypic information was available for 4 561 individuals.

### Statistical methods

Genome-wide association analysis was performed using PLINK (version 1.05) [35] and its additive linear regression model. To account for multiple testing, the generally accepted limit of P<5E-8 for genome-wide significance was used as a cut off value [36]. Further, the significance levels were evaluated using QQ plots. No notable deviation from the significance level expected by chance was observed (for further information see Figure S3). In addition to single SNPs, as an empirical limit for suggestive evidence of association, we considered clusters of ≥3 SNPs within a distance of 300 kb that associated with P<1E-4. These were considered as possible regions of interest and were followed by identifying the closest coding and non-coding genes located in UCSC Genome Browser (NCBI Build 36.1) (for details Supplementary Information). Coding genes in these regions of interest were analyzed for prior evidence for a role in psychiatric illness using UCSC Genome Browser (NCBI Build 36.1) and SZGene [37]. Further, a linkage disequilibrium analysis for the suggestive SNPs was performed using program Haploview [38]. The average r^2^of neighboring SNPs was 0.74 suggesting that they are relatively independent of each other and thus could be considered as internal replication of these regions, as a means to reduce the possibility of false positive observations.

### Pathway analysis

The relevance of the coding genes to the DISC1 pathway was studied through Ingenuity Pathway Analysis tools (IPA) (Ingenuity® Systems, www.ingenuity.com). All direct interactions between the identified genes with DISC1 and its IPA annotated interactors were studied. These interaction types are predominantly protein-protein binding and regulation of gene expression. When microRNAs were indentified, the predicted miRNA target sites were obtained through MicroCosm Targets (formerly miRBase Targets) [39]. This target gene list was then compared against IPA's own list of known genes to determine if there was significant enrichment in specific functions or pathways.

### Ethics statement

This study has followed the declaration of Helsinki and its amendments in full [40]. The study has been proved by Ministry of Social Affairs (Finland) and Ethical Committee of The Northern

Ostrobothnia Hospital District. All the participants in the study have provided their written informed consent.

## Supporting Information

Figure S1 **Manhattan plots for all the tested models:** RPAS risk (a), RPAS protective (b), RPAS neutral (c), RPAS covariated (d), RSAS risk (e), RSAS protective (f), RSAS neutral (g), RSAS covariated (h). Blue line corresponds to P = 10 E-4. The clusters with ≥3 SNPs with P<10E-4 are marked with asterisk (*).
(DOC)

Figure S2 **Illustration of how predicted target molecules of MIR620 that showed enrichment in bipolar disorder and psychological disorder have been related to etiologies of schizophrenia and psychosis.**
(DOC)

Figure S3 **The schematics show the QQ plots for the observed −log10 P-values versus those expected by change for Revised Social Anhedonia Scale.** (a) and Revised Physical Anhedonia Scale (b) risk (1), protective (2), neutral (3) and covariated (4) models.
(DOC)

Table S1 **Summary of the SNPs displaying P-values<10E-4 and located within clusters of three such SNPs. For all the SNPs, the closest gene and the shortest distance to the gene in base pairs are indicated.**
(XLS)

Table S2 **Summary of the SNPs with P<1E-4 within the loci with biological relevance for schizophrenia and the DISC1 pathway.**
(XLS)

Table S3 **Table of enriched IPA categories and functions for the gene targets of MIR620.**
(XLS)

Table S4 **Table of enriched IPA categories and functions for the gene targets of MIR-128-1.**
(XLS)
