# Supplementary material for: DISC1 Conditioned GWAS for Psychosis Proneness in a Large Finnish Birth Cohort
Source: PLoS One. 2012 Feb 17;7(2):e30643. doi: 10.1371/journal.pone.0030643 (PMC3281861; doi:10.1371/journal.pone.0030643)

Figure S1. Manhattan plots for all the tested models: RPAS risk (a), RPAS protective (b), RPAS neutral (c), RPAS covariated (d), RSAS risk (e), RSAS protective (f), RSAS neutral (g), RSAS covariated (h). Blue line corresponds to P = 10 E-4. The clusters with ≥ 3 SNPs with P < 10E-4 are marked with asterisk (*****).

a) *** ***


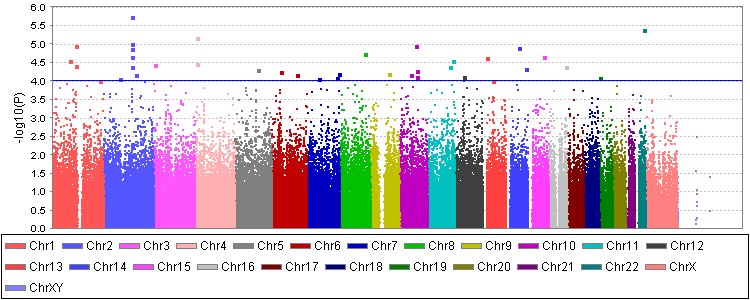


b)  ******


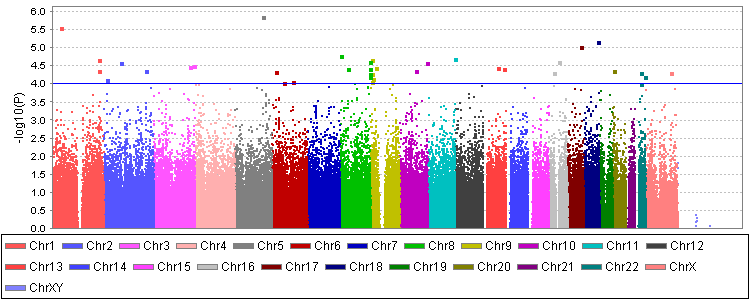


c) *** ***


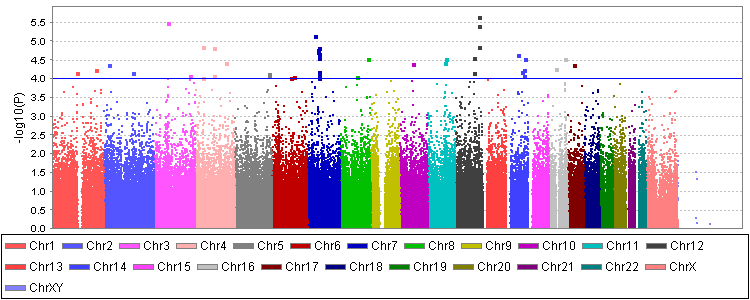


d) *** * * ***


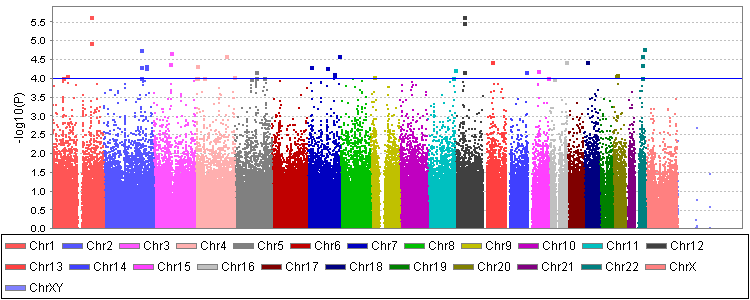


e) *****


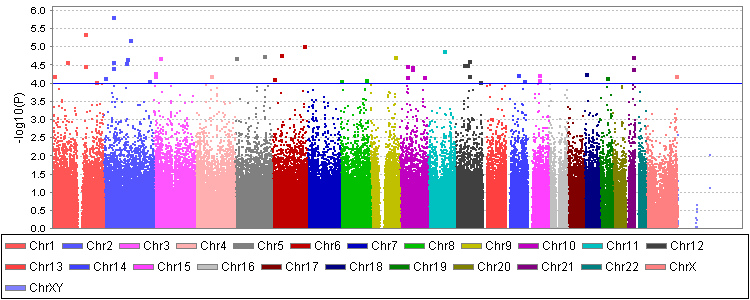


f) *** ***
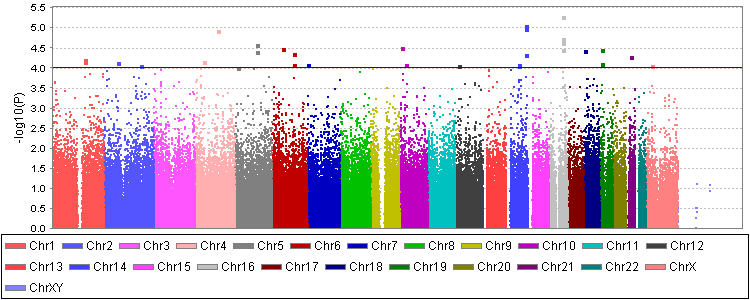


g)


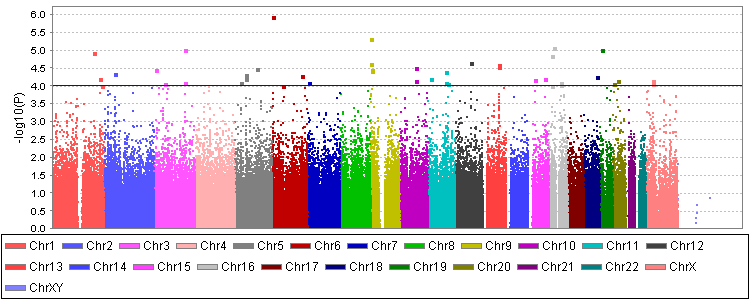


h) *** * * * ***


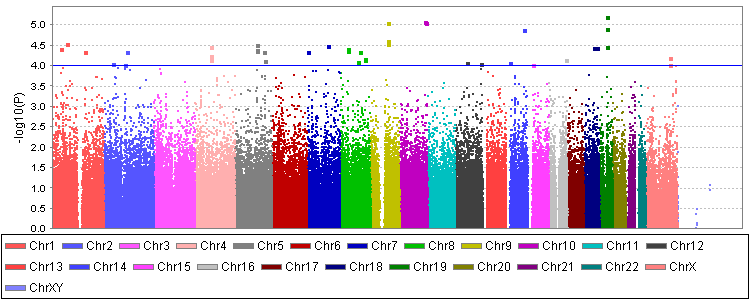


Color key:


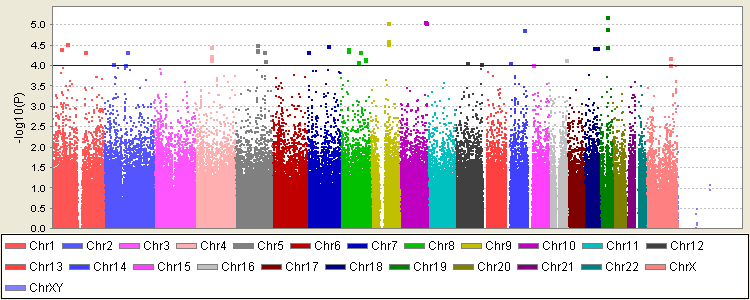

Supplement: Figure S1 — Manhattan plots for all the tested models: RPAS risk (a), RPAS protective (b), RPAS neutral (c), RPAS covariated (d), RSAS risk (e), RSAS protective (f), RSAS neutral (g), RSAS covariated (h). Blue line corresponds to P = 10 E-4. The clusters with ≥3 SNPs with P<10E-4 are marked with asterisk (*). (DOC) [file pone.0030643.s001.doc]
