# Supplementary material for: DISC1 Conditioned GWAS for Psychosis Proneness in a Large Finnish Birth Cohort
Source: PLoS One. 2012 Feb 17;7(2):e30643. doi: 10.1371/journal.pone.0030643 (PMC3281861; doi:10.1371/journal.pone.0030643)

Figure S2. Illustration of how predicted target molecules of MIR620 that showed enrichment in bipolar disorder and psychological disorder have been related to etiologies of schizophrenia and psychosis.


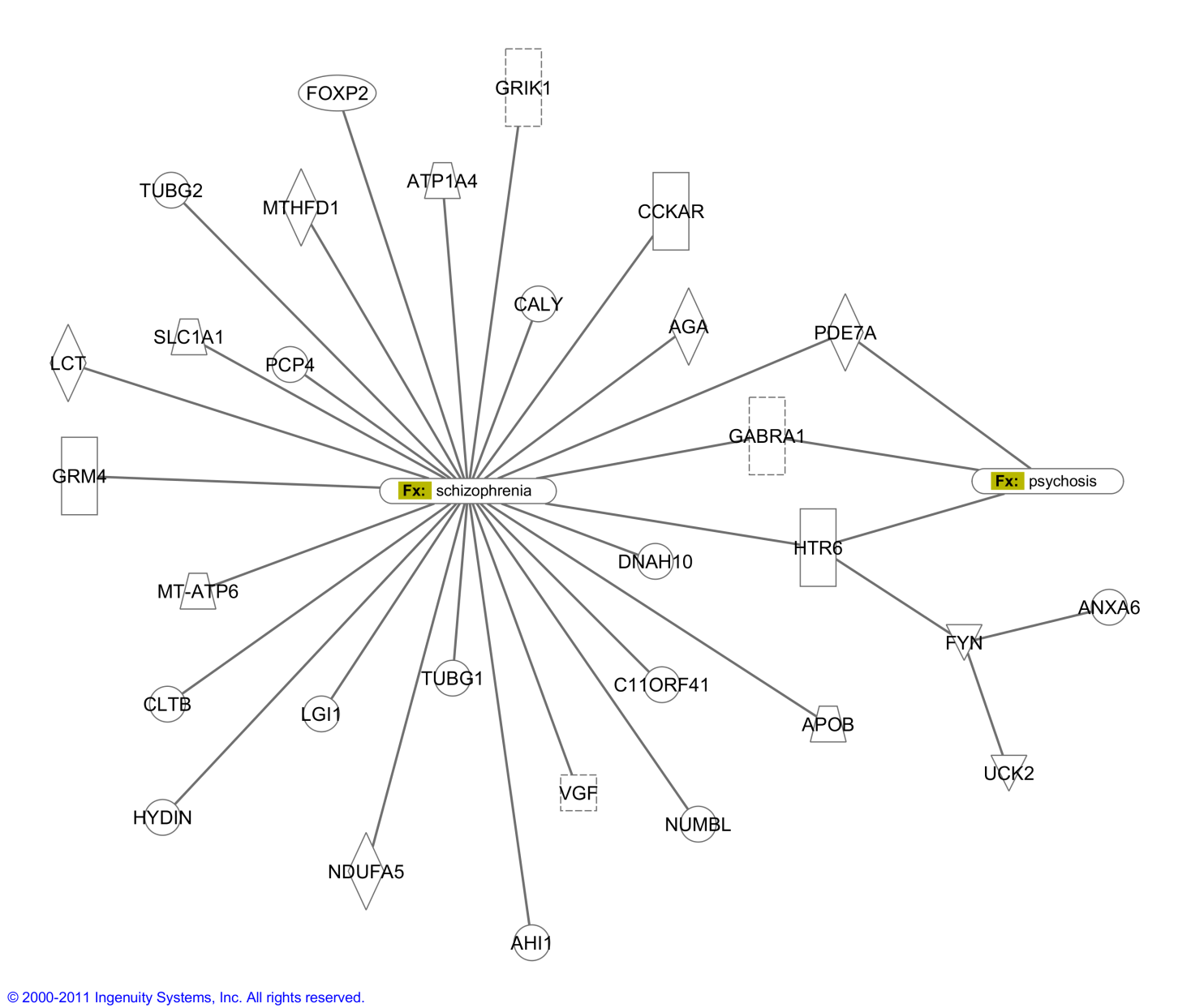

Supplement: Figure S2 — Illustration of how predicted target molecules of MIR620 that showed enrichment in bipolar disorder and psychological disorder have been related to etiologies of schizophrenia and psychosis. (DOC) [file pone.0030643.s002.doc]
