# Supplementary material for: DISC1 Conditioned GWAS for Psychosis Proneness in a Large Finnish Birth Cohort
Source: PLoS One. 2012 Feb 17;7(2):e30643. doi: 10.1371/journal.pone.0030643 (PMC3281861; doi:10.1371/journal.pone.0030643)
Supplement: Figure S3 — The schematics show the QQ plots for the observed −log10 P-values versus those expected by change for Revised Social Anhedonia Scale. (a) and Revised Physical Anhedonia Scale (b) risk (1), protective (2), neutral (3) and covariated (4) models. (DOC) [file pone.0030643.s003.doc]

Figure S3. The schematics show the QQ plots for the observed –log10 P-values versus those expected by change for Revised Social Anhedonia Scale (a) and Revised Physical Anhedonia Scale (b) risk (1), protective (2), neutral (3) and covariated (4) models.

a)

1) 2) 3) 4)

b)

1) 2) 3) 4
